# Cresyl Formothymol in the Treatment of Caries of the Third and Fourth Degree

**Published:** 1909-11-15

**Authors:** V. E. Miegeville, T. L. Larsenuer

**Affiliations:** Paris, France


### CRESYL FORMOTHYMOL IN THE TREATMENT OF CARIES OF THE THIRD AND FOURTH DEGREE.

BY DR. V. E. MIEGEVILLE, PARIS, FRANCE.

Translated from L'Odontologie by T. L. Larsenuer, D.D.S.

Among the numerous products which are utilized in the treatment of carious teeth of the fourth degree those which have Formol as their base certainly give the best results.

The commercial solution of formic aldehyde 40 per cent. strength is extremely antiseptic, more so than corrosive sublimate, according to M. Trillat; in all cases it is admitted that 25 centigrammes, that is to say about 5 drops of a 40 per cent. solution of the commercial drug, suffice to sterilize a litre of culture broth crowded with microbes, even if pathological ones. In the gaseous state it is the most energetic of known antiseptic agents; its vapor acts in the surest and most rapid manner, killing the most resisting pathological microbes with their spores. In order for this sterilization to be efficacious it is necessary for the formol or its vapor should be in direct contact with every part of the object to be disinfected.

Since in dental practice one cannot employ it in the gaseous state (there is certainly the aspiratory method proposed last year by one of our colleagues, but I must be very careful lest I be understood to regard it as practicable) it is necessary to employ it in solution, at the same time exercising a rational choice among those available. Practically the formol of commerce, which is a 40 per cent. aqueous solution of formic aldehyde presents serious inconveniences, which for the treatment of caries condemn it; it is its want of difusibility due to its slight volatility.

It is well known that if an aqueous solution of formol be carried to the boiling point, it volatilizes slightly, the aqueous vapor chiefly distills carrying off with difficulty (*a peine*) the formic aldehyde, which, when the point of concentration exceeds 50 per cent., remains at the bottom of the retort and becoming thicker little by little, gives rise by condensation to a complex compound body, solid and insoluble, composed in great part of trioxymethylene.

It was originally proposed to add in the presence of alcohol essence of geranium, thinking that an essence would make the formol diffuse more readily in the dental tissues, and likewise attenuate its irritant properties, but this addition of an essence sufficiently feebly antiseptic, diminished the concentration, and consequently the microbicidal power of the product, while communicating to it an odor disagreeable for the mouth.

One has then, in order to obtain a slight amount of diffusibility, reduced the antiseptic power of formol by essence of geranium; if one must make an addition to the formic aldehyde, in order to permit it to diffuse, it is necessary to at least add a product possessing real antiseptic power, above all having a solution absolutely anhydrom. Likewise, if the end in view was to attenuate the irritative property of the formal it was necessary, therefore, to add to it a product possessing analgesic properties, the addition of which does not sensibly diminish its bactericidal power.

It is after taking into account all these considerations that I made trial of essence of thyme containing 50 per cent. of thymol, a product strongly antiseptic, of which, according to M. Miguel, 2 grammes suffice to sterilize one litre of culture broth crammed with bacteria; ten times less toxic than phenol, to which it offers a complete analogy, it arrests putrid fermentation, and leaves the organic tissues unaltered.

Thymol by itself has already been employed by some practitioners for the treatment of infected canals; besides this, it was a product with an odor agreeable to the mouth, which entered into the composition of numerous dentifrices.

Finally after asepsis it was necessary to secure the greatest possible diffusibility by having a product which had great affinity for water; it was also absolutely indicated to utilize, as a dehydrant, chemically pure methylic alcohol, which, distilling at 65.5 degrees C, is more soluble than ethylic alcohol which only distills at 78.4 degrees C. One can then obtain, by means of special laboratory apparatus, a real saturation, by nascent formic aldehyde, cooled by the essence of thyme; the action of this product, already very antiseptic, can be reinforced by the addition of trikresol.

The method of operating is somewhat complicated, and necessitates great care; it is also advisable to prepare only small quantities at a time. A flask half filled with methylic alcohol is heated in a water bath (*bain-marie*), the alcohol evaporates, and, mixed with plenty of air, passes along a copper tube which contains coke heated to redness, without ever being carried to the point of incandescence, in order to avoid the production of carbonic acid. Formic aldehyde vapor mixed with that of methylic alcohol undecomposed passes off to be condensed in a U tube of large caliber filled one-fourth full of essence of thyme of known strength The tube is submerged in a receiver full of water, which is kept at a sufficiently low temperature by means of pieces of ice in summer time, or simply running water in winter. The operation is stopped when the volume is augmented by 50 per cent., at this moment the U tube can be replaced by a new one. A small measuring-bottle (*flacon de controle*) completes the apparatus.

The essence of thyme saturates itself with formic aldehyde mixed with alcohol methylic. I then add to the product obtained 20 per cent. trikresol, which is a mixture of three cresols roughly in the proportion of 25 paracresol, 35 orthocresol, and 40 metacresol. The idea of adding this last product is to considerably augment the antiseptic power of the constituents, chiefly by preserving their properties. The product thus obtained is absolutely anhydrous, unalterable, very limpid, and possesses considerable microbicidal power; its constitution is crysol-formo-thymol—that is to say, it is composed of trikresol, formol and thymol.

All these researches have been made specially for the benefit of dental practice, and, after numerous practical experiments extending over five years, I am able to say that the cresyl-formo-thymol has rendered me excellent service in numerous cases.

It is employed upon twist-drills for cases of the fourth degree, even the most highly infected, after mechanical cleaning, washing and complete drying of the cavity. Its use can also be extended with advantage to the making of an antiseptic paste for use under a permanent stopping, either in filling canals in cases of the fourth degree and in total pulpectomies.

The following formula for this paste gives good results, and replaces advantageously iodoform paste, of which the odor is so disagreeable both for the dentist and patient alike:

Formo-thymol, 1 to 4 drops.
Oxide of zinc.
aa. q. s. to make a firm paste.
Eugenol.

In pulpal amputations, the following is my method of operating: After an application of arsenic, cocaine pressure anaesthesia or an injection of novocaine into the gums, I clear the pulpal chamber of its contents, and after washing it out with tepid boiled water, followed by alcohol and chloroform, I dry it and insert cotton-wool soaked in cresyl-formo-thymol, which I leave twenty-four hours under gutta percha. At the next sitting I remove all this dressing, dry again, and fill the pulp chamber with the above mentioned paste. I cover this with cement, and after waiting from eight to ten days to see how the case goes on, I insert a permanent filling. In operating in this way with care, I have always good results.

In ordinary cases of the fourth degree, one or two dressings generally suffice to obtain a cure, three or four for stubborn and complicated cases.

Briefly, cresyl-formo-thymol, notwithstanding its antiseptic power and its great diffusibility, does not cause alve olo-dental inflammation; it rapidly permeates into the root-canals, impregnates the dry dentine and renders it sterile, at the same time speedily destroying the infective agents which have been able to find a home in it.—*American Dental Journal.*

